# A Comprehensive Study on the Anti-cancer Effects of Quercetin and Its Epigenetic Modifications in Arresting Progression of Colon Cancer Cell Proliferation

**DOI:** 10.1007/s00005-023-00669-w

**Published:** 2023-02-20

**Authors:** Meenu Bhatiya, Surajit Pathak, Ganesan Jothimani, Asim K. Duttaroy, Antara Banerjee

**Affiliations:** 1 Department of Medical Biotechnology, Faculty of Allied Health Sciences, Chettinad Academy of Research and Education (CARE), Chettinad Hospital and Research Institute (CHRI), Kelambakkam, Tamil Nadu 603 103 India; 2 Department of Nutrition, Faculty of Medicine, Institute of Basic Medical Sciences, University of Oslo, Oslo, Norway

**Keywords:** Anti-aging, Apoptotic, Anti-cancer, Colon cancer, Quercetin, Therapy

## Abstract

Colon cancer etiology involves a wide spectrum of genetic and epigenetic alterations, finding it challenging to find effective therapeutic strategies. Quercetin exhibits potent anti-proliferative/apoptotic properties. In the present study, we aimed to elucidate the anti-cancer and anti-aging effect of quercetin in colon cancer cell lines. The anti-proliferative effect of quercetin was assessed in vitro by CCK-8 in normal and colon cancer cell lines. To check the anti-aging potential of quercetin, collagenase, elastase, and hyaluronidase inhibitory activity assays were performed. The epigenetic and DNA damage assays were performed using the human NAD-dependent deacetylase Sirtuin-6, proteasome 20S, Klotho, Cytochrome-C, and telomerase ELISA kits. Furthermore, the aging-associated miRNA expression profiling was performed on colon cancer cells. The treatment with quercetin inhibited cell proliferation of colon cancer cells in a dose-dependent manner. Quercetin arrested colon cancer cell growth by modulating expression of aging proteins including Sirtuin-6 and Klotho and also by inhibiting telomerase activity to restrict the telomere length which is evident from qPCR analysis. Quercetin also exhibited DNA damage protection by reducing proteasome 20S levels. The miRNA expression profiling results displayed differential expression of miRNA in colon cancer cell, and in addition, the highly upregulated miRNA was involved in the regulation of cell cycle, proliferation, and transcription. Our data suggest that quercetin treatment inhibited cell proliferation in colon cancer cells through regulating the anti-aging protein expression and provides better understanding for quercetin’s potential use in colon cancer treatment.

**Supplementary Information:**

The online version contains supplementary material available at 10.1007/s00005-023-00669-w.

## Introduction

Colon cancer is the third most common leading cause of cancer-related death worldwide (Xi and Xu 2021). Genetic and epigenetic abnormalities highly influence colon cancer origin and progression. Despite significant advances in modern colon cancer treatments, such as surgical resection, radiotherapy, and chemotherapy, patients still need more advanced Prognosis and newer treatment strategies (Najafi et al. 2021; Podda et al. 2021). Surgical resection in colon cancer patients is a complex procedure with a greater risk of side effects such as pain, excessive bleeding, slow recovery, and recurrent infection (Varela et al. 2021). Likewise, chemotherapeutic drugs adversely affect the patient’s bone marrow, hair follicles, kidneys, lungs, and nervous system.

Apoptosis, or programmed cell death, is an energy-dependent molecular pathway. Apoptotic dysfunction is involved in the progression of colon cancer and its resistance to chemotherapeutic drugs and radiotherapy (Patra et al. 2021). A recent report states that genomic instability and epigenetic alterations are the major mechanisms of the malignant transformation of the colon (Grady 2021). Adenomatous polyposis coli, p*53*, and the proto-oncogene *Bcl-2* are the tumor-suppressor genes and oncogenes commonly associated with apoptosis and the development of colon cancer. However, the current treatment strategies are insufficient to prevent colon cancer progression and development. Hence, recurrent illness, poor response to therapy, and side effects underline the need for better and more sophisticated alternative treatments (Ionna et al. 2021).

Flavonoids, the dietary polyphenol extracted from vegetables and fruits, have been widely investigated for consumption that reduces the risk of cancer development, including colon cancer (Villota et al. 2022). Previous evidence suggests that polyphenol family members can modulate various signaling pathways by influencing the gene expression involved in cell cycle regulation, differentiation, and apoptosis (Almatroodi et al. 2021). Preliminary findings on applying natural products with augmented data suggest anti-proliferative potential in cancer cells versus normal cells. Potential natural compound constituents (flavonoid, polyphenol) may adversely affect apoptosis and cell cycle by modulating signal transduction pathways. Quercetin is a plant pigment, extracted from vegetables and fruits having high anti-oxidant, anti-inflammatory, and anti-proliferative activity (Datta et al. 2022). Quercetin’s anti-inflammatory activities have been explored in many in vitro and in vivo studies (Boly et al. 2011; Jiang et al. 2022). In addition, many researchers have fascinated attention to quercetin as an anti-inflammatory plant product since it exerts specific effects only on cancer cells rather than on normal and non-transformed cells (Bhatiya et al. 2021).

Cancer treatment is quite inadequate, resulting in poor effects due to disease recurrence. As a result, developing new therapeutic techniques is still a priority in the ongoing war against colon cancer. The primary aim of this study was to investigate the anti-cancer and anti-inflammatory potentials of quercetin and also to explore its role as an effective epigenetic regulator in primary and metastatic colon cancer cells. This study is undertaken to gain knowledge of the precise flavonoid-mediated epigenetic alterations which is essential for advancement in the formulation of novel therapeutic approaches against colon cancer.

## Materials and Methods

### Cell Lines and Chemicals

In our study, we used the following cell lines: LI32 (normal epithelial cell line), HCT 116 and COLO 320 (primary colon cancer cell line), and COLO 205 (metastatic colon cancer cell line). All cell lines were purchased from National Center for Cell Science, Pune, Maharashtra. The reagents MTT and DMSO were purchased from Sigma-Aldrich Ltd. (India). Quercetin (Cat. No- Q4951) was brought from Sigma-Aldrich Ltd. (India). Trypsin and cell culture medium and antibiotic solution were bought from GIBCO, Thermofisher (USA).

### Methods

#### Cell Culture and Cell Treatment

Normal lung epithelial and colon cancer cell lines were cultured in 20% DMEM supplemented with 10% fetal bovine serum and 1% antibiotic (100 IU/mL penicillin and streptomycin) at 37 ºC in normoxic conditions. For the cell treatment, 4 × 10^4^ cells were seeded into a T-25 flask and treated with selected doses of quercetin (80 µM and 120 µM) and incubated for 72 h (3 days). Thereafter, the cell lysate were isolated from treated cells and stored at – 20 °C for further studies.

#### Preparation of Quercetin Stock Solution and Working Solution

For the 20 mM quercetin stock solution preparation, 0.030 g of the quercetin was dissolved in 500 μl of dimethyl sulfoxide (DMSO) which is used as a solvent, volume makeup to 5 ml was done by adding autoclaved Milli-Q-water and stored at 4 °C. For treatment, various concentrations of working solution of quercetin as per the specific doses were directly taken from quercetin stock. To equalize the DMSO (solvent) effect, the control cells were supplemented with an equal proportion of DMSO (that was used to dilute the quercetin working solution) along with DMEM + FBS media.

#### The Experimental Groups

The experimental groups were as follows:

1. L132 (control)—L132 normal epithelial cells supplemented with DMEM + FBS media + DMSO (solvent);
2. L132-Q_80µM_ (LQ_80µM_)—L132 normal epithelial cells treated with 80 µM of quercetin;
3. L132-Q_120µM_ (LQ_120µM_)—normal epithelial cells treated with 120 µM of quercetin;
4. HCT 116 (control)—colon cancer cells supplemented with DMEM + FBS media + DMSO (solvent);
5. HCT 116-Q_80µM_ (HQ_80µM_)—colon cancer cells treated with 80 µM of quercetin;
6. HCT 116-Q_120µM_ (HQ_120µM_)—colon cancer cells treated with 120 µM of quercetin;
7. COLO 320 (control)—colon cancer cells supplemented with DMEM + FBS media + DMSO (solvent);
8. COLO 320-Q_80µM_ (C320Q_80µM_)—colon cancer cells treated with 80 µM of quercetin;
9. COLO 320-Q_120µM_ (C320Q_120µM_)—colon cancer cells treated with 120 µM of quercetin;
10. COLO 205 (control)—metastatic colon cancer cells supplemented with DMEM + FBS media + DMSO (solvent);
11. COLO 205-Q_80µM_ (C205Q_80µM_)—Metastatic colon cancer cell line treated with 80 µM of quercetin;
12. COLO 205-Q_120µM_ (C205Q_120µM_)—Metastatic colon cancer cell line treated with 120 µM of quercetin.

#### Cell Viability Analysis

##### CCK-8 Assay

The normal and colon cancer cells were seeded into 96-well plates at 2 × 10^3^ cells/well cell density and kept overnight at normoxic conditions (37 °C with 5% CO_2_). The medium was replaced with fresh medium containing various concentrations of quercetin (20, 40, 80, 120, 160, 320 µM) and incubated for 72 h. The cell proliferation ability or percentage of growth inhibition was determined using Cell Counting Kit-8 (Sigma-Aldrich, UA), a sensitive colorimetric assay kit, that has a higher detection sensitivity than other methods (MTT, XTT, or MTS). After treatment with quercetin working solution for 72 h, 10 μl of CCK-8 reagent was added and cells were incubated for 4 h. The difference in optical absorbance was measured at 450 nm by the ELISA plate reader (Robonik readwell touch ELISA plate reader). The IC_50_ (50% growth inhibitor concentration) value was calculated as the concentration of the quercetin that reduced cell viability by 50% as compared to untreated cells. The experiment was done in triplicates. The optimal dose with the least toxicity was used for further assays in all other cell lines.

#### Assessment of Cell Morphology Before and After Quercetin Treatment

To assess cell morphology before and after quercetin treatment to cells, all cells were seeded at 4 × 10^3^ cells/well in 24-well plates. Next day the medium was replaced with a medium containing quercetin (80 µM, 120 µM) and the treatment was done for 72 h. The cell morphology was assessed at 24, 48, and 72 h intervals, respectively. An inverted phase-contrast microscope was used to visually examine cell morphology during the quercetin treatment. Images were captured by an Inverted Leica Optica Image Viewer software.

#### Assessment of Anti-aging Property

##### Collagenase Inhibition Activity

Collagenase is a metalloproteinase that can degrade intermediate filament collagen. The measurement of the inhibition of collagenase was done with spectrophotometer, with some modifications (Van et al. 1981). The reaction mixture of Tricine buffer (50 mM), CaCl_2_ (10 mM), NaCl (400 mM) pH 7.5 was prepared. Collagenase from *Clostridium histolyticum* (0.01 U/ml in the cool equates), sample, and reaction mixture was incubated for 20 min at 37 °C. Then, the synthetic substrate *N*-[3-(2-furyl) acryloyl]-Leu-Gly-Pro-Ala (FALGPA) was added to the final reaction mixture. The absorbance was measured at 335 nm after 20 min of adding substrate in spectrophotometer (Shimadzu UV Spectrophotometer, USA). The experiment has been performed in a triplicate manner.

##### Elastase Inhibition Activity

Elastase is a metalloproteinase that degrades molecules such as elastin intermediate protein. The elastase inhibition activity was measured by standard protocol. Briefly, a reaction mixture was prepared with elastase substrate (N-Sucanyl-Ala-Ala-Ala-p-Nitroanilide, Cat. no. 54760; Sigma, USA), Tris–HCL buffer (2 mM, pH 8), sample (30 µl) and incubated for 15 min at 25 °C. After incubation, 20 µl of 3 U/ml porcine pancreatic elastase (Sigma, USA) was added to the reaction mixture and co-incubated for 15 min at 25 °C. The optical density was measured at 410 nm by spectrophotometer. The experiment has been performed in a triplicate manner.

##### Hyaluronidase Inhibition Activity

Hyaluronidase inhibition activity was evaluated as described by Sahasrabudhe and Deodhar (2010) with few modifications. First, an enzyme solution with sodium phosphate buffer (20 mM, pH 7.0), sodium chloride (77 mM), bovine serum albumin (BSA; 0.01%, pH 7.0), and 1.50 U hyaluronidase (hyaluronidase from bovine testes, Cat. no. H0779; Sigma, USA), was prepared. The enzyme solution (100 μl) and sample (10 μl) were incubated for 10 min at 37 °C. Following incubation, 100 µl of 0.03% hyaluronic acid (HA) salt (hyaluronic acid sodium salt, Cat. no. 75810, SRL) prepared in 20 mM sodium phosphate buffer (pH 5.0) were incubated at 37 °C for 45 min. After incubation, the unabsorbed HA was precipitated with 1 ml of 0.1% BSA (prepared in 24 mM sodium acetate and 79 mM acetic acid, pH 3.75). Thereafter, the reaction solution was incubated at room temperature (RT) for 10 min. At 600 nm, the absorbance was measured. The percentage of hyaluronidase inhibition activity was calculated (%) = (1 − *B*/*A*) × 100, where *A* is the enzyme activity without the sample and *B* is the activity with the sample.

#### ELISA Assays for Analyzing Aging-Related Protein Expression

##### Assessment of Human Klotho Protein Expression

The human Klotho protein expression assay was performed using the human Klotho ELISA Kit (Elabscience, Cat No. E-EL-H5451) protocol. The various standard diluents (10, 5, 2.5, 1.25, 0.63, 0.31 ng/ml) were made by serially dilution of reference standard (20 ng/ml). The working solution (100 µl), sample, and sample diluent were added into the standard, sample and blank wells, respectively. At 37 °C, the solution was incubated for 1 h 30 min. After incubation, the surplus solution has been replaced with Biotinylated Detection Ab (100 µl) and incubated at RT for 1 h. Following three washes, HRP conjugate (100 µl) was added to the well followed by 30 min at 37 °C. After incubation, the wells were washed five times. The substrate solution (90 µl) was added to the well. The incubation was performed in dark for 15 min at RT. A stop solution (50 µl) was added to pause the reaction. A microplate reader was used to measure the optical density (OD) at 450 nm.

##### Assessment of NAD-Dependent Deacetylase Sirtuin-6 Expression

The NAD-dependent deacetylase Sirtuin-6 expression was measured using the ELISA Kit (Bioassay Technology Laboratory, Cat No. E2562Hu) and was performed according to the manufacturer’s instructions. Five standard diluents (24, 12, 6, 3, 1.5 ng/ml) were prepared by serial dilution of standard stock solution (48 ng/ml). Standard (50 µl) was added into standard wells S1 to S5, respectively. In the sample and control wells, 40 µl of sample and 10 µl of anti-SIRT-6 antibody were added, followed by 50 µl of streptavidin-HRP. The mixture was incubated for 1 h at RT. After incubation, the wells were washed five times with wash buffer. Then, substrate solution A (50 µl) and substrate solution B (50 µl) were added to each well and incubation have been performed in dark for 10 min at 37 °C. After incubation, a stop solution (50 µl) has been added to stop the reaction. The OD has been measured at 450 nm with the help of a microplate reader.

#### Assays Depicting DNA Damage

##### Assessment of Human Cytochrome-C

The Human Cytochrome-C (Cyt-C) ELISA Kit (Abbkine, Cat No. KTE62179) was used to analyze the level of Cytochrome-C expression. 50 µl of Standard and sample were added into standard and sample wells, respectively. The plate was incubated at RT for 45 min. After incubation, the wells were gently washed five times with wash buffer. After washing, HRP-conjugated Ab (50 µl) was added to all wells except blank and incubated for 30 min incubated at 37 °C. The washing step was repeated. After washing, 50 µl of chromogen A and chromogen B were added and incubated in the dark for 15 min at RT. To halt the reaction, 50 µl of stop solution was added to the reaction mixture. Using a microplate reader, OD was recorded at 450 nm.

##### Assessment of Human Proteasome 20S

The 20S Proteasome expression in colon cancer and normal epithelial cells was analyzed using ELISA Kit (Human 20S Proteasome, Abbkine, Cat No. KTE62530). 50 µl of Standard and sample with added into standard and sample wells, respectively. At 37 °C, the reaction mixture was incubated for 45 min. After incubation, the wells were washed with wash buffer. After washing, 50 µl HRP-conjugated detection-Ab was added into each well except blank and kept for 30 min incubation at 37 °C. After incubation, the wells were washed five times with wash buffer. After washing, 50 µl of chromogen A and chromogen B was added to the wells. The reaction mixture has been incubated in dark for 15 min at 37 °C. After incubation, 50 µl of stop solution has been added to the reaction mixture and the absorbance (OD) was recorded by a microplate reader at 450 nm within 15 min.

#### Epigenetic Modulation Assessment

##### Assessment of Human Telomerase

The expression level of telomerase in the colon cancer cells and normal epithelial cells was determined using the Human telomerase ELISA Kit (Bioassay Technology Laboratory, Cat No. E2562Hu). The assay follows the kit protocol. 50 µl of Standard was added into respective standard wells S1–S5, respectively. 40 µl of sample and 10 µl of anti-telomerase antibody were added to the sample well and 50 µl of streptavidin-HRP was added to the sample as well as standard wells (except blank and control well). The plate was incubated for 1 h at 37 °C. After incubation, the wells were washed with wash buffer. After washing, 50 µl of substrate solution A and substrate solution B was added to the reaction mixture. The reaction mixture was then incubated in the dark for 10 min at RT. After incubation, to halt the reaction, a stop solution (50 µl) was added to the reaction mixture, and the absorbance was evaluated within 15 min at 450 nm with the help of a microplate reader.

##### Relative Human Telomere Length Quantification qPCR Assay

The DNA was isolated from LI32, HCT 116, COLO 320, and COLO 205 cells. Telomere length quantification was done using the RHTLQ qPCR assay kit (Catalog no. #8908, Science Cell Research Laboratories). Telomere primers are used to recognize and amplify telomere sequences. The single-copy reference primer set recognizes and amplifies a 100-bp region on human chromosome 17 which is used as a reference for data normalization.

#### Aging-Related miRNA Screening

The total RNA was isolated from COLO 320, and COLO 205 using a Qiazol lysis reagent (Qiagen, USA). The RNA concentration and purity was estimated using Nanodrop. MiR cDNA was synthesized using 200 ng of RNA, the miScript RT II kit (Qiagen, USA) following the manufacturer’s protocol. The miRNA array (qPCR-based) was performed on synthesized cDNA using pre-coated miRNA array plates (MIHS 122ZD—human stem cell, Qiagen, USA) and miScript SYBR green master mix (Qiagen, USA). The target miRNA quantification cycle (*Cq*) values were normalized with the *Cq* values of the reference miRNAs to calculate Δ*Ct* and the fold change (2^–Δ*Ct*^) was calculated and the graphs were plotted using Graphpad Prism V9.

#### Bioinformatical Analysis of microRNA–mRNA Interaction and Functional Enrichment Analysis

Functional enrichment analysis was performed on clustered miRNAs in a database based on their association with the GENE of interest. Here, we selected the top 20 highly expressed miRNAs from COLO 320 primary and COLO 205 metastatic colon cancer cells for enrichment analysis. In miRNet V2.0, a list of miRNA names has been given as a query. *Homo sapiens*, miR Base ID, and Genes from miRTarBase V8.0 are the query parameters for the selection. The network was then curated using the network tool “degree filter”. The degree filter’s cutoff value was set to 7.0 to reduce network size and identify key hubs. Finally, miRNA functional enrichment analysis was carried out to determine the biological function of co-expressed hubs, such as miRNA molecular function and its associated gene ontology (GO), Kyoto Encyclopedia of Genes and Genomes (KEGG) pathway analysis, GO reactome analysis, and GO biological process. Graphpad Prism V9 was used to create the graphs.

### Statistical Analysis

The mean ± SEM of data were obtained from three independent experiments. To determine whether there were significant differences between two datasets, control and treated samples, the unpaired *t* test (two-tailed) has been used. The statistical significance in the graph, (*) indicates a *p* value less than 0.05 (*p* < 0.05), (**) indicates a *p* value less than 0.01 (*p* < 0.01), (***) indicates a *p* value less than 0.001 (*p* < 0.001). In the data, the control (untreated) group of the respective cell lines has been compared with the quercetin-treated groups in each group. The GraphPad V9 software was used to analyze all the data.

## Results

### Assessment of Cell Morphology After Quercetin Treatment

**Fig. 1:**
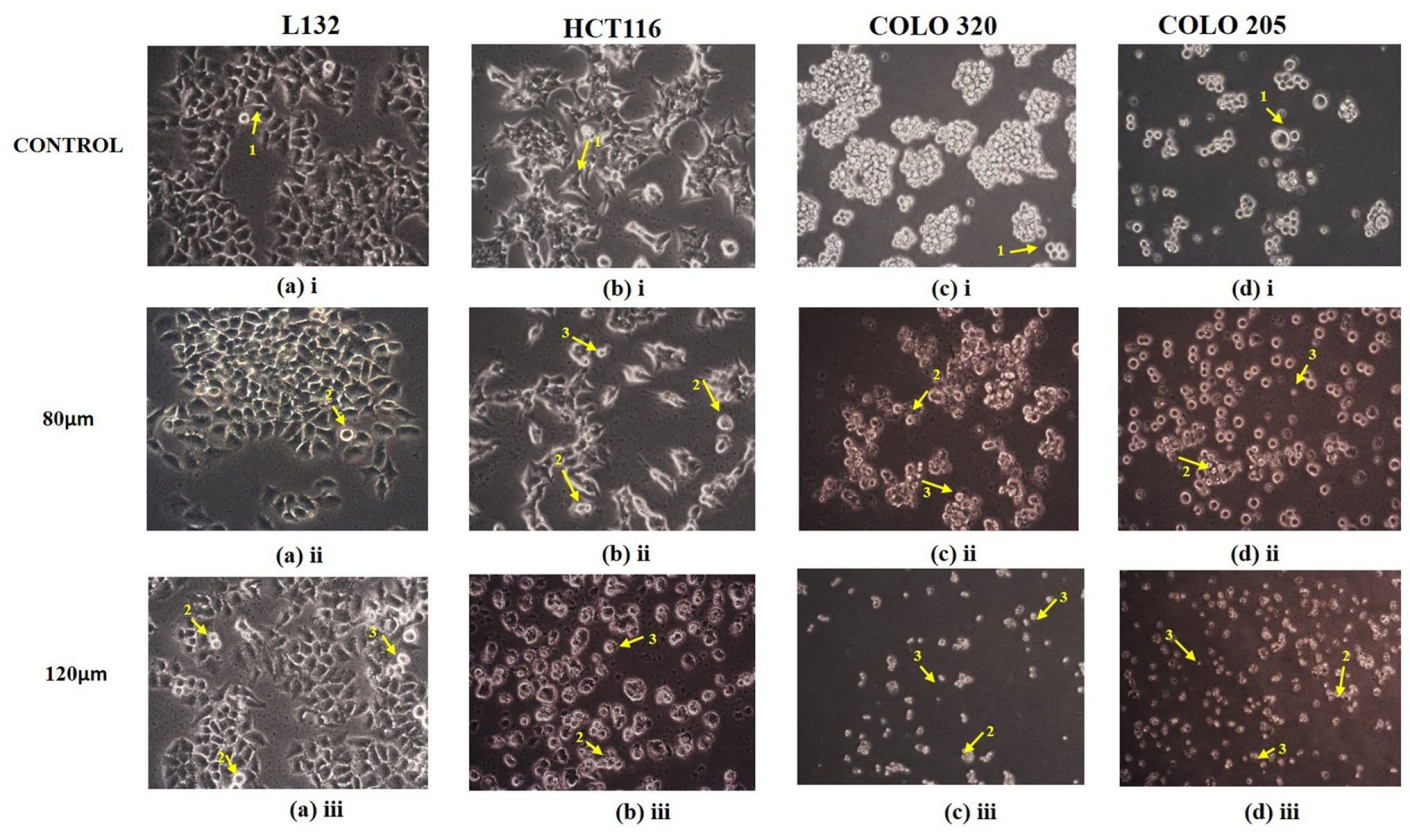
Representative image shows morphological changes of selected cell lines: **a** (i–iii) L132 Normal epithelial cell line, **b** (i–iii) HCT 116 primary colon cancer cell line, **c** (i–iii) COLO 320 primary colon cancer cell line, **d** (i–iii) COLO 205 metastatic colon cancer cell line. Cells were treated with quercetin for 3 days and imaged by an inverted phase-contrast microscope (magnification 10×). (1) Normal cell morphology, (2) apoptotic bodies, and (3) cell shrinkage are indicated by arrows

The cell morphology was assessed at 24, 48 and 72 h intervals, respectively. Significant morphological alterations were observed during the treatment in HCT 116, COLO 320, and COLO 205 cells, whereas no significant morphological changes were observed in LI32. The HCT 116, COLO 320, and COLO 205 cells were observed with reduced proliferation potential compared to untreated cells. Visualization of the control HCT 116 cells showed that cells maintained their original morphology, exhibited elongated projections, and adhered to the disc’s surface, whereas HCT 116 cells were treated with quercetin, they changed their fibroblastic appearance, and revealed apoptotic features such as round and shrunk cell shape due to loss of adherent property with adjacent cells and to the surface, babbling of membrane, and nuclear condensation was observed. In contrast, no significant changes were observed in L132 normal epithelial cells. Likewise, COLO 320, and COLO 205 control cells have enlarged rounded morphology with colonies that become shrunken when treated with quercetin. The changes in morphology after treatment are depicted in Fig. 1.

### Cell Viability Analysis

#### CCK-8 Assay

**Fig. 2:**
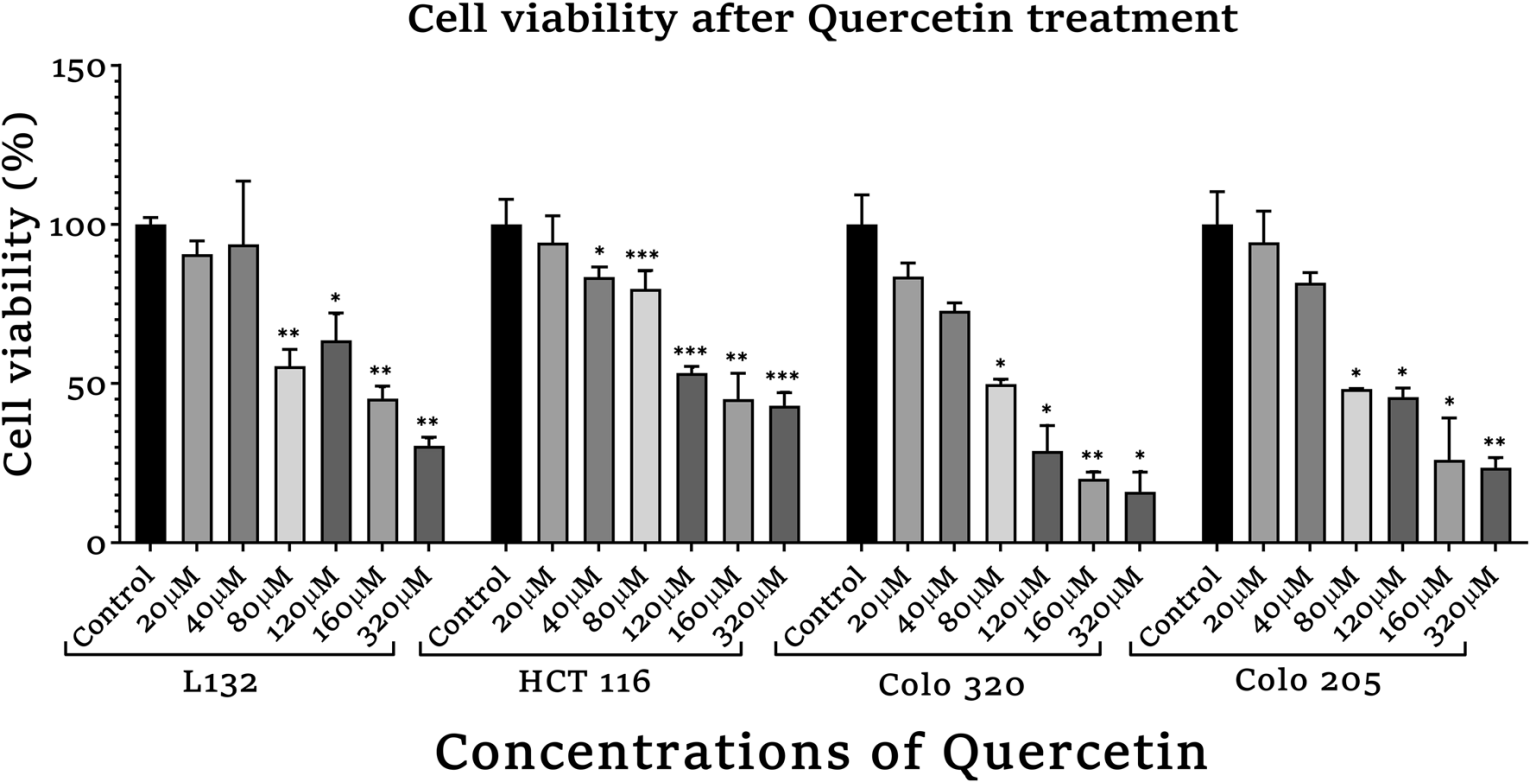
Cell viability analysis of quercetin-treated normal, primary and metastasis colon cancer cells by CCK-8 assay. The statistical significance in the graph indicates *p < 0.05; **p < 0.01; ***p < 0.001

The CCK-8 proliferation assay was carried out in control as well as colon cancer cells to identify whether quercetin influences the survival of the cells and to calculate the IC_50_ value of quercetin for cell viability estimation and also based on previous studies conducted in our laboratory to molecularly characterize primary and metastatic colon cancer cells with natural compounds including quercetin as therapeutic targets through in vitro studies and bioinformatics (Jothimani et al. 2022; Oliveira et al. 2020). The cells were exposed to different concentrations of quercetin as shown in Fig. 2. The analysis was done based on control cells supplemented with medium + FBS + DMSO (solvent). After 72 h, we observed that the quercetin showed high inhibition of cell viability in a dose and time-dependent manner with IC_50_ values of 80 µM in L132 cells and 120 µM in all the colon cancer cell lines. Since in 160 µM and 320 µM concentrations, the growth of cells were heavily hindered in all cell lines, hence 80 µM and 120 µM of quercetin treatment were used for all cell lines for further experiments.

### Anti-aging Assays

#### Collagenase Inhibition Activity

**Fig. 3:**
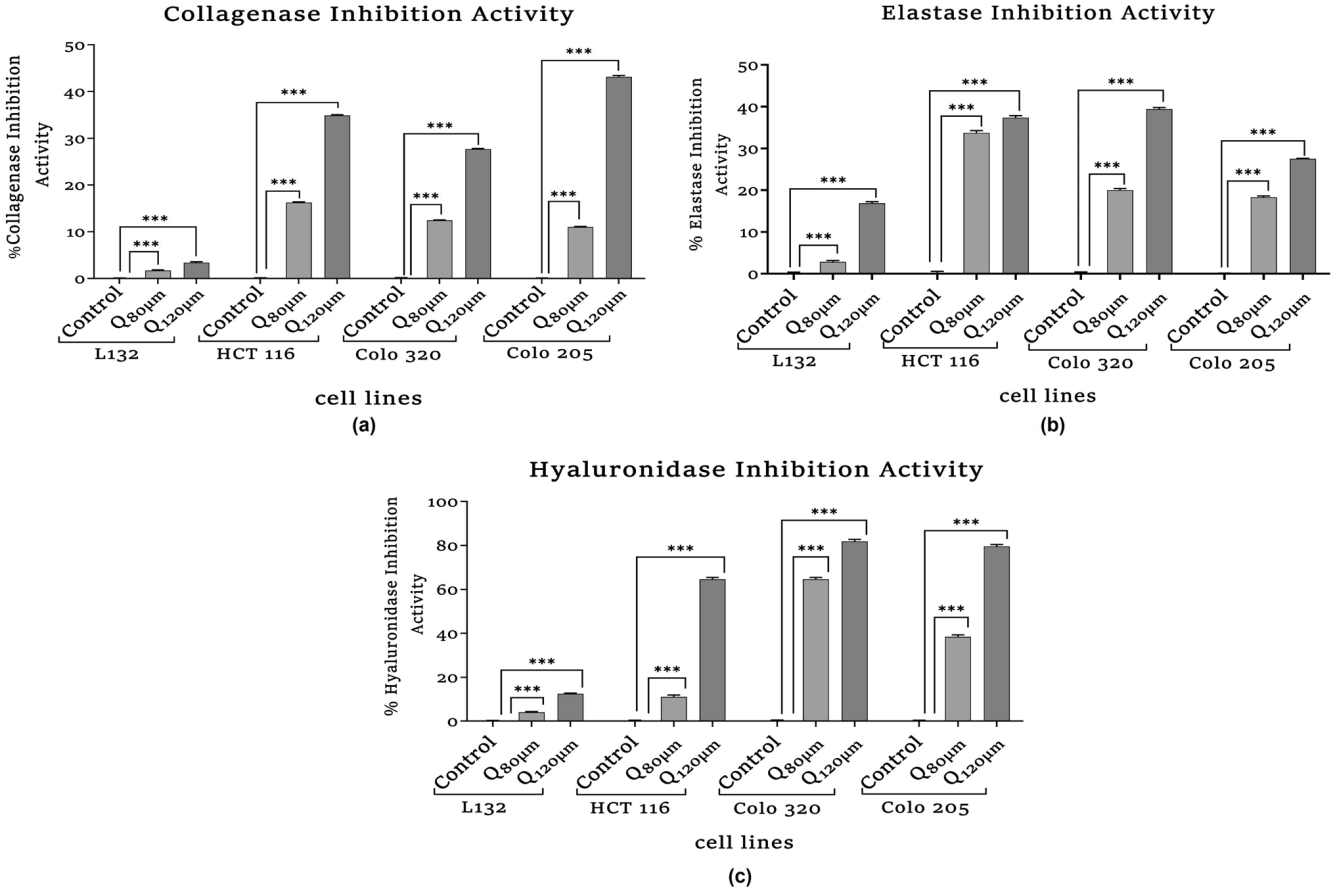
Assays were performed to measure inhibitory effect of quercetin on aging-related enzymes. **a** Collagenase inhibitory activity. **b** Elastase inhibitory activity. **c** Hyaluronidase inhibitory activity was measured by spectrophotometric method. The statistical significance in the graph, indicates *p < 0.05; **p < 0.01; ***p < 0.001

The collagenase inhibition activity is elucidated as shown in Fig. 3a. Quercetin's effect on collagenase inhibition was notable in the cancer cell lines. The collagenase inhibitory activity was significantly increased in HCT116 (HQ_120µM_, ***p = 0.001), C320Q_120µM_ (***p = 0.001), and C205Q_120µM_ (***p = 0.001) when compared with their respective controls.

#### Elastase Inhibition Activity

The elastase inhibition activity is represented in Fig. 3b. The effect of quercetin treatment on various cell lines was observed to be significantly increasing. In HCT 116 (HQ_80µM_, HQ_120μM_, ***p < 0.001), COLO 320 (C320Q_80µM_, C320Q_120µM_, ***p < 0.001) and COLO 205 (C205Q_80µM_, C205Q_120µM_, ***p < 0.001), the significant rise when compared with their respective controls was observed.

#### Hyaluronidase Inhibition Activity

The hyaluronidase inhibition activity is elucidated as shown in Fig. 3c. Significant hyaluronidase inhibition was observed in quercetin-treated HQ_80µM_ (***p < 0.001) and HQ_120µM_ (***p < 0.001) compared to the HCT 116 untreated group. Similarly, in COLO 320 cells, significant hyaluronidase inhibition is observed in both C320Q_80µM_ (***p < 0.001) and C320Q_120µM_ (***p < 0.001) when compared to the COLO 320 control group. A similar trend was also observed in the metastatic cells in quercetin-treated C205Q_80µM_ (***p < 0.001) and C205Q_120µM_ (***p < 0.001), whereas in normal L132, a significant change is observed but the range inhibitory activity was very less.

### Aging-Related Protein Expression Analysis Through ELISA

#### Assessment of Human Klotho Expression

**Fig. 4:**
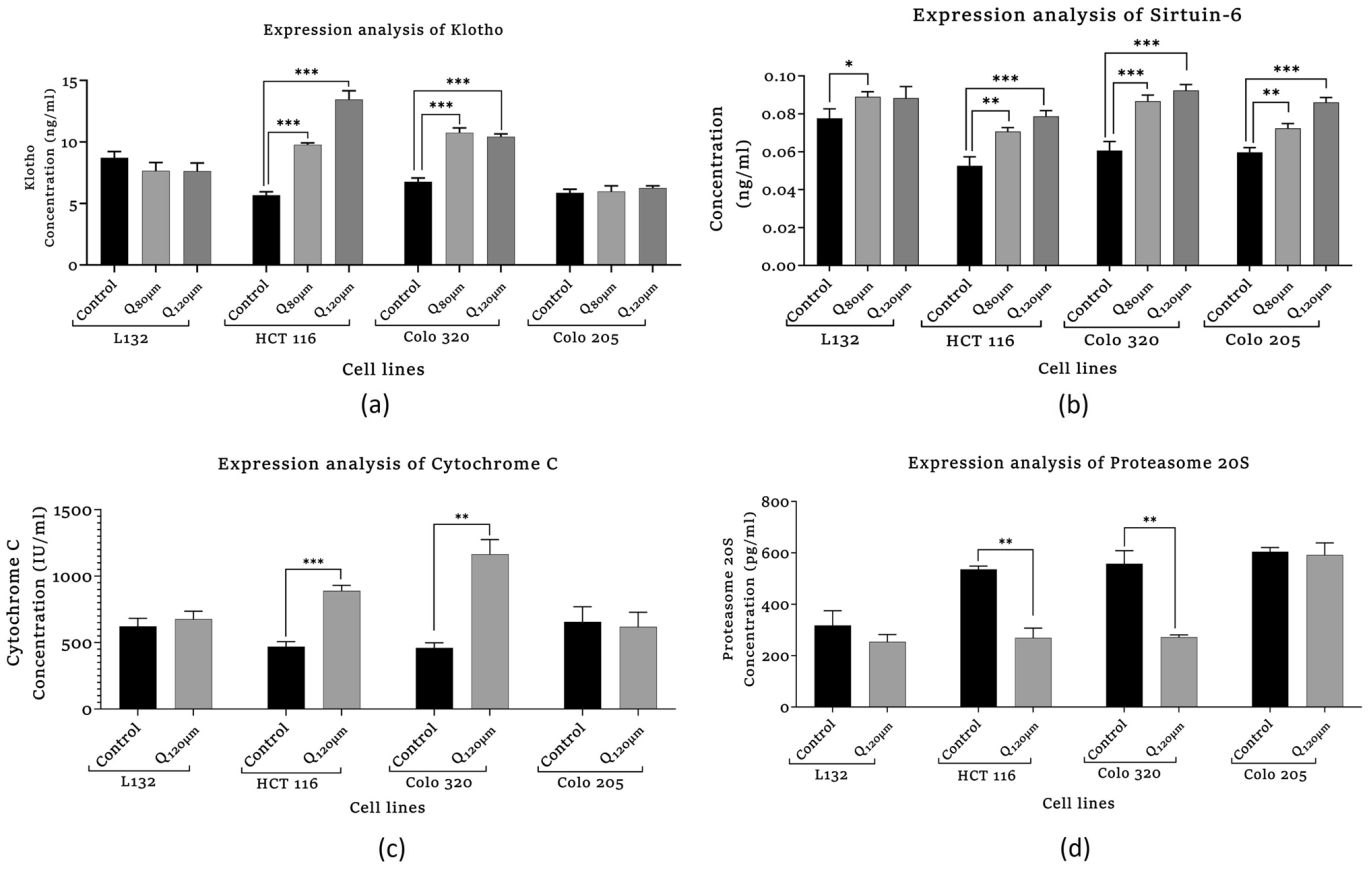
Effect of quercetin on aging-related protein expression in quercetin-treated and control, primary and metastasis colon cancer cells. **a** Human Klotho expression. **b** NAD-dependent deacetylase Sirtuin-6. DNA damage assay. **c** Human Cytochrome-C assay. **d** Human proteasome 20S assay. The statistical significance in the graph indicates *p < 0.05; **p < 0.01; ***p < 0.001

The expression level of Klotho was seen to be significantly increasing in quercetin-treated colon cancer cells (Fig. 4a), In HCT 116, the expression of Klotho was significantly increased in HQ_80µM_ (***p = 0.001) and HQ_120µM_ (***p < 0.001) compared to the HCT 116 control group. Likewise, the Klotho expression in COLO 320 was significantly increased in both C320Q_80µM_ (***p = 0.001) and C320Q_120µM_ (***p < 0.001) treated groups as compared to the COLO 320 and control group, whereas quercetin-treated COLO 205 cells and normal L132 cells had no changes.

#### Assessment of Human NAD-Dependent Deacetylase Sirtuin-6 (SIRT-6) Expression

The expression of SIRT-6 was significantly increased in quercetin-treated L132 cells (LQ_80 µM_, *p = 0.02) and HCT 116 cells (HQ_120µM_, ***p = 0.001). As shown in Fig. 4b, COLO 320 (C320Q_120µM_, ***p = 0.001), and COLO 205 cells (C205Q_120µM_, ***p = 0.001) as compared with their respective controls a similar trend was observed. In HCT 116, the expression of SIRT-6 is significantly more in HQ_120µM_ (***p = 0.001) and HQ_80µM_ (**p = 0.004) as compared to the HCT 116 control group. The same trend was observed in COLO 205, where the expression of SIRT-6 is significantly more in C205Q_120µM_ (***p < 0.001) and C205Q_80µM_ (**p = 0.004) as compared to the COLO 205 control group. In COLO 320 cells, the expression of SIRT-6 was significantly increased in both C320Q_80µM_ (***p = 0.001) and C320Q_120µM_ (***p < 0.001) as compared to the COLO 320 control group.

### DNA Damage Assays

#### Assessment of Cytochrome-C Expression

The expression of Cytochrome-C was significantly increased in quercetin-treated cells. The quercetin-treated HCT 116 cells (HQ_120µM_, ***p = 0.001), COLO 320 cells (C320Q_120µM_, **p = 0.01) showed significant increase as compared with their respective control group, whereas in the COLO 205 and L132 cells, there was no significant difference observed in Cytochrome-C expression before and after the treatment (Fig. 4c).

#### Assessment of Proteasome 20S

The expression of Proteasome 20S was significantly increased in quercetin-treated colon cancer cells, whereas no significant change was observed in L132 cells. The quercetin-treated HCT 116 and COLO 320 cells showed a decline in Proteasome 20S activity (HQ_120µM_, **p = 0.009), (C320Q_120µM_, **p = 0.01) significantly as compared with their respective control groups, whereas in COLO 205 group, there was no significant change observed (Fig. 4d).

### Epigenetic Modulation Assessment

#### Assessment of Human Telomerase Expression

**Fig. 5:**
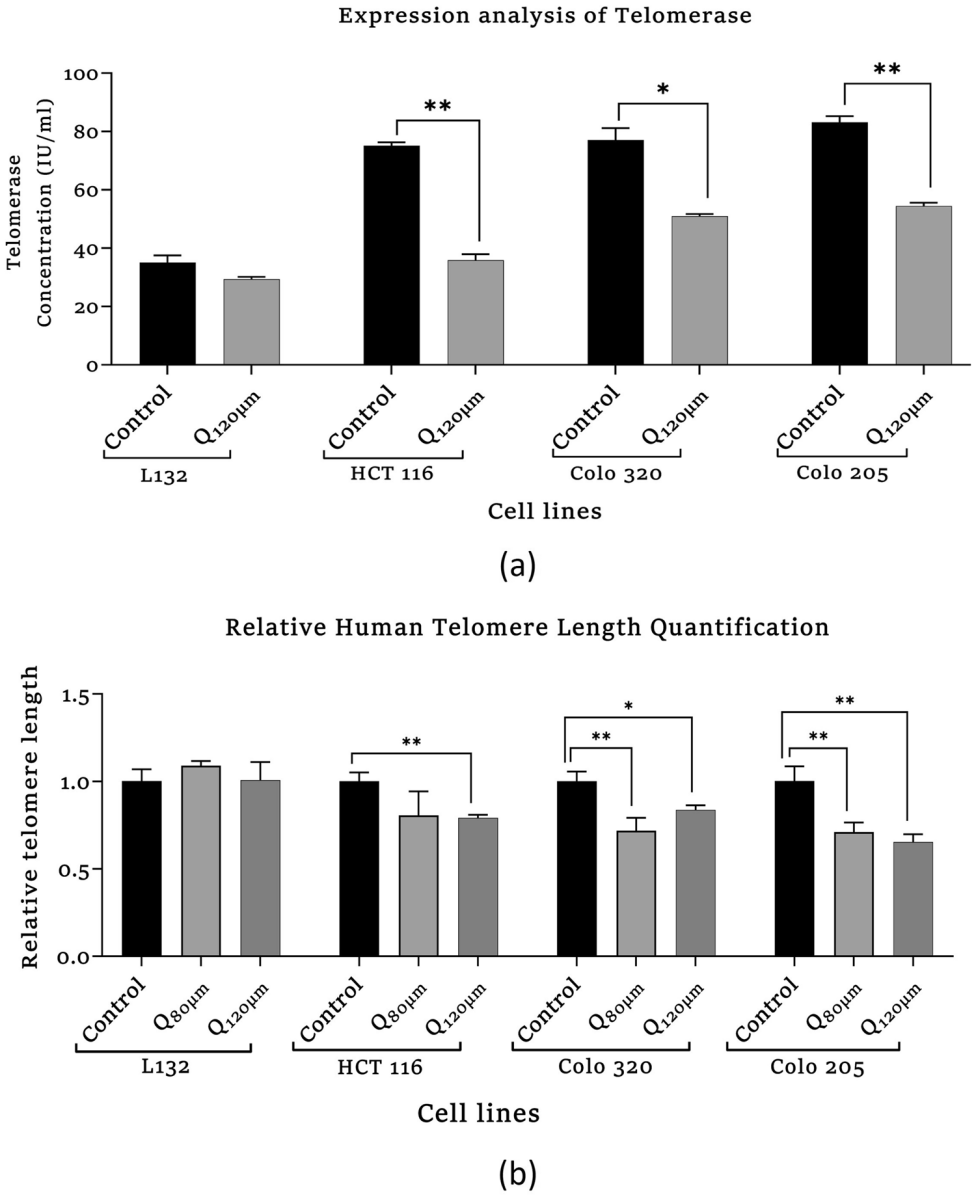
Analysis of epigenetic modulatory effect of quercetin by **a** Human Telomerase enzyme (TE) ELISA. **b** Relative telomere length quantification of quercetin-treated and control, primary and metastasis colon cancer cells. The statistical significance in the graph indicates *p < 0.05; **p < 0.01; ***p < 0.001

Results showed that the expression of telomerase was significantly decreased in colon cancer cells which are elucidated in Fig. 5 (a). In quercetin-treated HCT 116 and COLO 205 cells, the expression of telomerase was more significantly decreased (HQ_120µm_, **p = 0.002) and (C205Q_120µm_, **p = 0.003), respectively, as compared to their respective control group. Likewise, in the COLO 320 cell, telomerase expression decreased significantly (C320Q_120µm_, **p = 0.01) compared to the COLO 320 control group, whereas there was no significant change observed in L132 cells.

#### Relative Human Telomere Length Quantification qPCR Assay

The number of telomeres repeats amplification product (T) compared to a single-copy gene (S) product is used to calculate telomere length. The T/S ratio is related to telomere length on average. As presented in Fig. 5(b), the telomere length was significantly decreased in quercetin-treated colon cancer cells, whereas no significant change was observed in L132 cells. The HCT 116 cells (HQ_120µM_, **p = 0.002) and COLO 205 cells showed (C320Q_120µM_, **p = 0.01), (C320Q_80µM_, **p = 0.006) significant decrease in telomere length as compared with their respective control groups. COLO 205 (C205Q_80µM_, **p = 0.003, C205Q_120µM_, **p = 0.007) showed a significant decrease in the telomere length as compared with their respective control groups.

### Anti-aging-Related miRNA Array Analysis

**Fig. 6:**
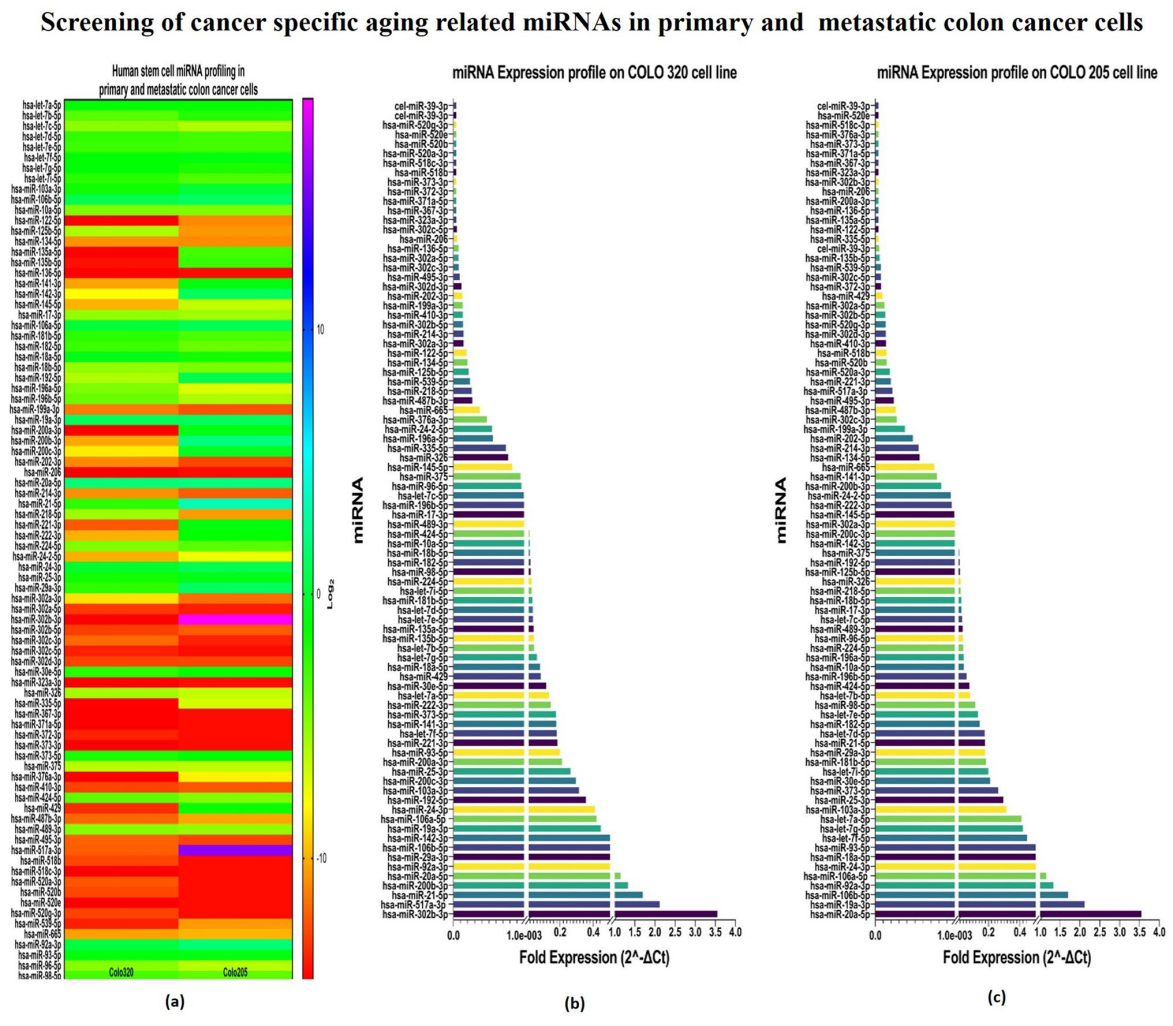
Aging-related miRNAs expression profiling in primary and metastatic colon cancer. **a** Heat map presentation, **b** Aging-related miRNAs fold expression in primary (COLO 320) and, **c** in metastatic (COLO 205) colon cancer cells

The miRNA expression profiling had shown that *hsa-mir-21, hsa-mir-20a, hsa-mir-92a-1, hsa-mir-92a-2, hsa-mir-29a, hsa-mir-106b, hsa-mir-19a, hsa-mir-106a, hsa-mir-24–1, hsa-mir-24–2, hsa-mir-103a-2, hsa-mir-103a-1, hsa-mir-25, hsa-mir-93, hsa-let-7f-1, hsa-let-7f-2, hsa-mir-373, hsa-let-7a-1, hsa-let-7a-2, hsa-let-7a-3, hsa-mir-30e, hsa-mir-18a,* and *hsa-let-7g* were upregulated in COLO 320 and COLO 205 cells which were involved in the regulation of cell cycle, cell differentiation, migration, and invasion; whereas miRNAs such as *hsa-mir-539, hsa-mir-122, hsa-mir-302a, hsa-mir-302b, hsa-mir-410, hsa-mir-302d, hsa-mir-302c, hsa-mir-136, hsa-mir-206, hsa-mir-323a, hsa-mir-371a, hsa-mir-372, hsa-mir-373, hsa-mir-518b, hsa-mir-518c, hsa-mir-520a, hsa-mir-520b, hsa-mir-520e, hsa-mir-520g*, and *hsa-mir-367* were extremely downregulated/less expressed in both primary and metastatic colon cancer cells. Interestingly, we found a signature pattern of increased expression of miRNAs including *hsa-miR-302b-3p, hsa-miR-517a-3p, hsa-miR-200a-3p, hsa-miR-200b-3p, hsa-miR-429, hsa-miR-135a-5p, hsa-miR-135b-5p, hsa-miR-221-3p, hsa-miR-141-3p, hsa-miR-142-3p, hsa-miR-222-3p, hsa-miR-200c-3p, hsa-miR-335-5p, hsa-miR-192-5p,* and *hsa-miR-376a-3p* only in metastatic colon cancer cells (COLO 205). Elucidating the role of these highly expressed miRNAs in metastatic cells might aid in the better understanding the progression of cancer from primary to metastasis stage. Consequently, this pattern of dysregulated miRNA expression provides numerous potential therapeutic targets, the most common of which it might be used in the treatment of cancer. These miRNAs may activate genes that inhibit tumor development or suppress oncogenes that are significantly downregulated in cancer cells (Fig. 6a–c). The differential miRNA expression in primary and metastatic colon cancer is shown in Supplementary Table 1.

### MicroRNA–mRNA Interactions and Functional Enrichment Analysis of Aging-Related, Highly and Differentially Expressed miRNAs in Colon Cancer Cell Lines

**Fig. 7:**
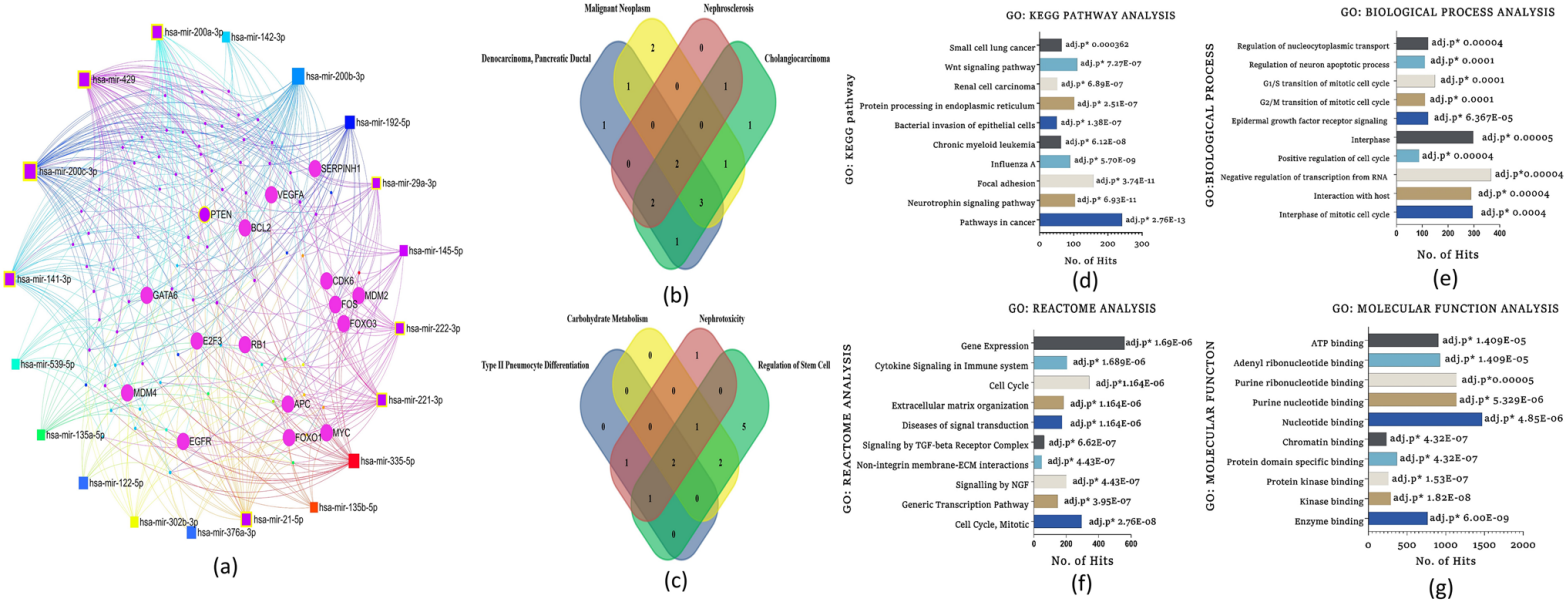
**a** miRNA–mRNA interactions network of highly expressed miRNAs in primary and metastatic colon cancer using the MiRNet database. **b** Venn diagrammatic representation of miRNAs distribution in functional enrichment analysis. **c** Venn diagrammatic representation of interacting nodes distribution in biological process analysis. **d** GO KEGG analysis of interacting nodes with highly expressed miRNAs with top ten pathway class having highly significant *p* value **e** GO biological process analysis of interacting nodes with highly expressed miRNAs with top ten pathway class having highly significant *p* value. **f** GO Reactome analysis of interacting nodes with highly expressed top ten miRNAs having highly significant *p* values. **g** Molecular functional analysis of highly expressed miRNAs with top ten function class having highly significant *p* value

The miRNA–mRNA network analysis classified miRNAs based on their biological function, molecular functions, and interaction, revealing that the majority of miRNAs were involved in apoptosis, signaling, cell proliferation, and aging with a highly significant adjusted *p*-value (Fig. 7). MiRNet and Funrich are the target gene prediction tools that are used to find miRNA-targeted genes. Approximately 4869 genes were identified as the target genes of 20 highly expressed miRNAs during the processes. To get the accuracy of the analysis, a Venn diagram is used to find common genes among the differentially expressed miRNAs and the 4869 target genes. Finally, we identified 458 shared genes. We perform GO analysis and KEGG pathway enrichment analysis to better understand the biological roles of these 458 common genes. According to the GO analysis, more than 439 genes are involved in the cellular component (CC), 415 genes in the biological process (BP) 312 genes in the cellular process (CP) category. The GO analysis and KEGG pathway enrichment analysis of miRNA and associated genes revealed a significant link to the cell growth and proliferation, and cell cycle regulators, suggesting that these top 20 abundant colon cancer miRNAs (COLO 320, COLO 205) may have tumor-suppressive properties and may influence future cancer therapy research.

## Discussion

Various factors are considered to be associated with colon cancer morbidity and mortality rates, for instance, western diet and lifestyle, inadequate physical exercise and weight gain, genetic alteration, and epigenetic modifications (Akimoto et al. 2021), whereas the accumulation of gene mutations and epigenetic alteration may influence the emergence and spread of non-neoplastic to neoplastic adenocarcinoma. Likewise, aging is also a progressive decline process with the accumulation of gene mutations and cellular-level epigenetic modifications, which may ultimately result in the development of aging-associated diseases including colon cancer (Zabransky et al. 2022). Quercetin, a glycoside, has a high medicinal value, involved in a variety of cellular activities including anti-aging, cardiovascular, anti-inflammation, and anti-cancer activities (Salehi et al. 2020; Sharma et al. 2021). Hence, quercetin among the flavonoids is thought to have potential bioactivity as medicinal usage for cancer therapy. Here, the experiment was carried out with quercetin to examine its anti-aging, apoptotic, anti-proliferation, and epigenetic modulatory properties in normal epithelial cells and in primary, and metastatic colon cancer cells.

In our study, CCK8 assay was performed to validate quercetin’s effect on the cell viability of normal and colon cancer cells where results showed a strong cytotoxic effect of quercetin in the normal and cancer cell proliferation. As shown in Fig. 2, the analysis showed that quercetin suppressed the cell viability in a dose-dependent manner in all the cell lines with significant differences. The results showed that the IC_50_ values of 80 µM in L132, COLO 320, COLO 205 and 120 µM in HCT 116 was observed. Since in higher concentrations of 160 µM and 320 μM quercetin treatment, the growth of cells were heavily hindered, hence 80 µM and 120 µM of quercetin doses were selected for the treatment of all cell lines for further experiments. In this study, the morphological alteration in cancer cells also has been demonstrated after quercetin treatment. As a result, the COLO 320, and COLO 205 colon cancer cells lost their spherical morphology and became shrunken. However, normal epithelial cells L132 were not seen much affected after quercetin treatment. The cell shrinkage and apoptotic bodies were also visible in the images. As we already mentioned above that, cancer and aging are both driven by the same principles: the accumulation of genetic alteration, gene mutation, DNA damage, and epigenetic modifications over time is one of factor in aging-related diseases and promotes cancer progression (Mejia-Ramirez and Florian 2020). Various extracellular matrix (ECM)-related studies have reported the involvement of major structural protein such as collagen and elastin in aging as well as cancer. The well-known enzyme collagenase and elastase break down collagen and elastin ECM filaments whose expression increases with aging (Karamanos et al. 2021). Similarly, in the case of cancer, collagenase and elastase indicate their role in tumor progression by facilitating tumor cell invasion and migration (Lepucki et al. 2022). In our results, we observed that collagenase and elastase inhibition were drastically increasing in quercetin-treated cells, proving quercetin’s anti-aging potential that strongly inhibits collagenase and elastase activity. Similarly, hyaluronidase is an endoglycosidase, that break-down hyaluronic acid into monosaccharides. Hyaluronic acid is a glycosaminoglycan, a major component of ECM. An increased level of HA is involved in cancer progression. In the study, hyaluronidase was found to be highly associated with more aggressive stages and involved in colon cancer progression (Chen et al. 2022). Quercetin effectively inhibited hyaluronidase activity in treated cells as well. Similar to HA, Klotho has been demonstrated to play a significant role in aging. However, in contrast to HA, Klotho expression facilitates the inhibition of various key pathways and limits the activity of proteins involved to promote tumorigenesis. A comprehensive dataset revealed that frequent hypermethylation of Klotho promoter results in poor expression of Klotho in colon cancer cells and suggests that DNA methylation alterations could serve as biomarkers for colon cancer. The precise impact of Klotho’s epigenetic alteration and overexpression may lead to cell proliferation inhibition, cell cycle arrest, and induction of apoptosis. The analysis of Klotho protein expression in our study revealed that quercetin stimulated the expression of Klotho in colon cancer cells, promoting tumor-suppressing activity. Since Klotho inhibits insulin/IGF1, p53/p21, and Wnt signaling pathway, it may be possible through the reports here to more thoroughly understand how Klotho functions as a tumor suppressor that might aid in developing more effective treatments strategies for colon cancer (Arbel Rubinstein et al. 2019). A relatively similar functional relationship has been observed between Klotho and SIRT-6. Both the proteins help prolong longevity and protect against aging-related diseases including cancer. In the present study, it was revealed that quercetin treatment showed a significant increase in SIRT-6 expression (a member of the evolutionarily conserved histone deacetylase sirtuin family, and also known to play a notable role as an epigenetic modifier by histone deacetylation, including the maintenance of chromatin structure and the regulation of transcription factor and epigenetic enzyme activity (Bosch-Presegué and Vaquero 2015)) in both primary and metastatic colon cancer cells. Furthermore, downregulated SIRT-6 expression has been identified in the early stages of human colon cancer and is maintained during tumor growth, suggesting that a low level of SIRT-6 may have been involved in the initiation and progression of colon cancer (Korotkov et al. 2021; Tian and Yuan 2018). A recent study had demonstrated that the transcriptional activity of HIF-1 and MYC is inhibited by overexpressed SIRT-6, which limits the EMP pathway and promotes cell cycle arrest (Zhang et al. 2019). SIRT-6 significantly promotes apoptosis pathways by deactivating the anti-apoptotic factor and stimulating p53 and p73 (Akter et al. 2021). Therefore, our data comprehend that SIRT-6 activation was required for apoptosis to occur in colon cancer cell lines. Apart from apoptosis, Cytochrome-C is involved in several cellular processes such as electron transfer, free-radical scavenging, apoptosome formation, and redox-coupled protein import (Lagoa et al. 2017). Our study results suggest that quercetin treatment induced Cytochrome-C upregulation and enhanced its expression level to promote apoptosis. Our finding indicates that with the release of Cytochrome-C, quercetin treatment also upregulating *p53* and *BAX* expression significantly whereas, reducing the expression of anti-apoptotic proteins (*Bcl-2 and Bcl-XL*) and promoting the intrinsic pathway of apoptosis (Srivastava et al. 2016). It already been reported that proteasomes are highly specialized protease complex that performs metabolic energy-dependent intracellular protein degradation. The protein degradation is initiated by the binding of a ubiquitin chain, which serves as a signal to transport the target proteins to the proteasome (Trulsson et al. 2022). On the basis of previous studies, it is well known that proteasome regulation is important for intracellular homeostasis as well as for the functioning of several signaling pathways. In the case of carcinogenesis, proteasome activity is usually increased which promotes carcinogenesis by providing anti-apoptotic protection and effective elimination of abnormal proteins in cancer cells (Soundararajan and Kim 2018). Furthermore, telomerase is a ribonucleoprotein complex enzyme responsible for telomere length maintenance by adding guanine-rich repetitive sequences at the end of the chromosomes (Srivastava et al. 2022). In colon cancer, high telomerase activity was demonstrated by high levels of hTERT promoter methylation. It is known that bioactive substance targets and modulates methylation at TERT. Lower methylation at hTERT promotes strongly associated with shorter telomeres and lower telomerase activity (Dogan and Forsyth 2021). Hence, the flavonoid anti-tumor activity alters the degree of methylation at the promoter region and thereby suppresses telomerase activity which leads to telomere shortening in cancer cells and approaching cellular senescence that leads to cell death which is inline with other reports (Zvereva et al. 2010). Here, in our experimental study quercetin treatment showed suppression in telomerase expression in all the colon cancer cell lines compared to their respective control groups. Hence, the study results emphasized that quercetin reduced cell viability by increasing apoptosis and necrosis and quercetin might potentially delay the progression and limit the proliferation of colon cancer cells.

Furthermore, studies from our team emphasizes that microRNAs can modulate specific miRNAs implicated in oncogenesis, progression, and metastasis also having pleiotropic effects on various pathways (Malayaperumal et al. 2020; Pathak and Banerjee 2021). The RISC (RNA-induced silencing complex) microRNA interaction recognizes its target sequence across mRNA and deactivates it via the RNA silencing mechanism. It cleaves RNA at the translated sequence or the 3′-UTR (Aubrey et al. 2018). In our study, the aging-related miRNA screening was aimed to investigate the alteration in miRNA expression levels from primary to metastatic stages, which might provide useful information about possible therapeutic targets and might highlight the insights of various miRNAs that are actively involved in metastasis’s immune evasion mechanisms (Malayaperumal et al. 2021). As per the results of the miRNA screening, miR-302b-3p, miR-517a-3p, miR-21-5p, miR-200b-3p, and miR-20a-5p were found highly upregulated and miR-39-3p, miR-520g-3p, miR-518c-3p, and miR-373-3p highly downregulated in COLO 320 primary colon cancer cell. In a previous study, the miR-302b-3p has been reported to function as a tumor suppressor which usually is downregulated in cancer. According to the previous finding, miR-302b-3p significantly inhibits cell proliferation by targeting IGF-R via its UTR through the AKT pathway.

Furthermore, it has been reported that miR-302b-3p also induces cell cycle arrest and apoptosis. According to recent bioinformatics analysis findings, high expression of miR-302b-3p was reported in aged skin cells and senescence cells. The findings also revealed that overexpressed miR-302b-3p plays a crucial role in skin aging by directly targeting the JNK2 gene (Tan et al. 2020). The tumor-associated miRNA miR-517a plays a key role in metastasis. In comparison to adjoining normal cells, miR-517a was found to be considerably higher in colon cancer cells. As a result, miR-517a could be used as a prognostic biomarker for colon cancer patients (Ma et al. 2016). In exosomes of colon cancer cells, miR-21-5p expression was shown to be significantly higher than in normal colon cells. Since miR-21-5p is involved in cell proliferation, invasion, and extracellular matrix, targeting it could be a novel approach for colon cancer treatment (Sun et al. 2020). Moreover, miR-21-5p is actively involved in the modulation of the NF-κB pathway, thereby associated with various inflammatory conditions and aging-related diseases (Olivieri et al. 2021). The miR-520 family is downregulated in colon cancer which suppresses tumor growth and metastasis. EGFR signaling plays an important role in colon cancer progression therefore, the EGFR pathway is an important inhibitor of tumor growth. It has been found that the miR-520 family is an upstream regulator of EGFR which could be a potential therapeutic approach by targeting this pathway (Zhang et al. 2017). In COLO 205 cells, miR-20a-5p, miR-19a-3p, miR-106b-5p, and miR-92a-3p were upregulated, while miR-39-3p, miR-520e, miR-518c-3p, and miR-376a-3p were downregulated. The loss of the Smad4 promoter accelerates the progression of colon cancer. The Smad4 protein is involved both as a transcription factor and a tumor suppressor. Recently, scientists found that certain miRNA directly regulates Smad4 expression in cancer aggression, and miR-20a-5p negatively regulates Smad4 in colon cancer by specifically targeting it's 3' UTR (Cheng et al. 2016). miR-19a-3p is actively involved in colon cancer proliferation, invasion, and migration of colon cancer by targeting T cell intracellular antigen 1; thus, it could represent a prognostic biomarker (Ardizzone et al. 2021). MiR-19a is a well-known oncogenic miRNA among the miR-19 family members, and its overexpression has been linked to cell proliferation, invasion, migration, metastasis, tumor size, stage of development, and poor prognosis (Deka et al. 2021; Jothimani et al. 2018). As a result, miR-19a is believed to be a potential biomarker and potential therapeutic target for diagnostic and prognostic applications. One method by which these flavonoids exert anti-cancer action could be possibly targeting human telomeric G-quadruplex DNA (Elibol and Kilic 2018). Telomerase length decreased significantly in quercetin-treated colon cancer cells, and telomere degradation. In summary, our studies illustrate that quercetin suppresses colon cancer cell proliferation by inhibiting telomerase activity by arresting telomere length in colon cancer cells. According to our findings, quercetin might have been a promising alternative for targeting the telomere and hence acting as an anti-cancer drug.

To better explore these miRNAs involvement in defining various cellular processes, we carried out GO function and KEGG pathway analysis. GO KEGG pathway analysis showed that the upregulated miRNAs were particularly enriched in cancer, neurotrophin signaling pathways, focal adhesion, and chronic myeloid leukemia. GO Reactome analysis showed that the upregulated DEmiRNAs were particularly enriched in cell cycle, mitotic, signaling by cytokine, NGF, TGF-β receptor complex, organization of extracellular matrix, and ECM interaction. GO biological processes analysis showed that the upregulated DEmiRNAs were particularly involved in the regulation of the cell cycle, nucleoplasmic transport, EGFR signaling pathway, interphase, G1/S, and G2/M transition of the mitotic cell cycle. GO molecular function analysis showed that the upregulated miRNAs were mainly involved in binding protein kinase, kinase, enzymes, chromatin binding, and ATP binding.

## Conclusion

The findings of this experiment indicate that quercetin could inhibit tumor cell proliferation. The results demonstrate that quercetin intake might have a significant role in increasing apoptosis, inhibiting metastasis, and cell cycle regulation. Further, quercetin also modulates the expression of anti-aging genes SIRT-6 and Klotho to inhibit colon cancer cell proliferation. The miRNA expression profile and functional enrichment analysis provided various molecular targets which might actively regulate cell proliferation, apoptosis and cellular senescence. In addition to the regulation of aging-associated miRNA and anti-aging protein activity such as Klotho and SIRT-6, inhibition of telomerase activity leading to the shortening of telomere length paved an interesting avenue towards future designing of colon cancer therapeutics. Thus, a combinational therapeutic regime using quercetin along with epigenetic modifiers with existing chemotherapeutic drugs might help to manage the disease progression of colon cancer and reduce the side effects of the chemotherapeutic drugs.

### Supplementary Information

Below is the link to the electronic supplementary material.

Supplementary file1 (DOCX 22 KB)

## Data Availability

All datasets emerging from the experiments which were analyzed during the current study have been represented in the figures in the manuscript and are also available with the corresponding authors.
