# Supplementary material for: A Comprehensive Study on the Anti-cancer Effects of Quercetin and Its Epigenetic Modifications in Arresting Progression of Colon Cancer Cell Proliferation
Source: Arch Immunol Ther Exp (Warsz). 2023 Feb 20;71(1):6. doi: 10.1007/s00005-023-00669-w (PMC9941246; doi:10.1007/s00005-023-00669-w)
Supplement: Supplementary file 1 — Supplementary file1 (DOCX 22 KB) [file 5_2023_669_MOESM1_ESM.docx]

**Supplemntary Table 1: Screening of cancer specific aging-related miRNAs in primary and metastatic colon cancer (COLO 320 and COLO 205):**

| **COLO 320** | | **COLO 205** | |
| --- | --- | --- | --- |
| **miRNA** | **2^-ΔCt** | **miRNA** | **2^-ΔCt** |
| hsa-miR-302b-3p | 436805.7805 | hsa-miR-302b-3p | 0.000040018 |
| hsa-miR-517a-3p | 54998.56178 | hsa-miR-517a-3p | 0.000215779 |
| hsa-miR-21-5p | 10.695292 | hsa-miR-21-5p | 0.177140411 |
| hsa-miR-200b-3p | 4.250478984 | hsa-miR-200b-3p | 0.000830295 |
| hsa-miR-20a-5p | 4.027949393 | hsa-miR-20a-5p | 3.546814135 |
| hsa-miR-92a-3p | 3.546793947 | hsa-miR-92a-3p | 1.331180461 |
| hsa-miR-29a-3p | 2.416493816 | hsa-miR-29a-3p | 0.179026472 |
| hsa-miR-106b-5p | 2.390012849 | hsa-miR-106b-5p | 1.697663364 |
| hsa-miR-142-3p | 2.279143141 | hsa-miR-142-3p | 0.004096569 |
| hsa-miR-19a-3p | 2.265403228 | hsa-miR-19a-3p | 2.116985617 |
| hsa-miR-106a-5p | 1.95864556 | hsa-miR-106a-5p | 1.147461732 |
| hsa-miR-24-3p | 1.807037975 | hsa-miR-24-3p | 0.932734218 |
| hsa-miR-192-5p | 1.655728197 | hsa-miR-192-5p | 0.017416335 |
| hsa-miR-103a-3p | 1.103470084 | hsa-miR-103a-3p | 0.313566076 |
| hsa-miR-200c-3p | 0.785408446 | hsa-miR-200c-3p | 0.002984768 |
| hsa-miR-25-3p | 0.617959661 | hsa-miR-25-3p | 0.294257801 |
| hsa-miR-200a-3p | 0.57804701 | hsa-miR-200a-3p | 0.000040018 |
| hsa-miR-93-5p | 0.556698234 | hsa-miR-93-5p | 0.527208662 |
| hsa-miR-221-3p | 0.541429178 | hsa-miR-221-3p | 0.000196235 |
| hsa-let-7f-5p | 0.508712033 | hsa-let-7f-5p | 0.445044978 |
| hsa-miR-141-3p | 0.483360785 | hsa-miR-141-3p | 0.000775605 |
| hsa-miR-373-5p | 0.441317233 | hsa-miR-373-5p | 0.262390158 |
| hsa-miR-222-3p | 0.436532929 | hsa-miR-222-3p | 0.000960018 |
| hsa-let-7a-5p | 0.380522633 | hsa-let-7a-5p | 0.409055216 |
| hsa-miR-30e-5p | 0.372387901 | hsa-miR-30e-5p | 0.210611624 |
| hsa-miR-429 | 0.338287961 | hsa-miR-429 | 0.000088694 |
| hsa-miR-18a-5p | 0.289998831 | hsa-miR-18a-5p | 0.63341884 |
| hsa-let-7g-5p | 0.261602867 | hsa-let-7g-5p | 0.418977111 |
| hsa-let-7b-5p | 0.219014417 | hsa-let-7b-5p | 0.082635923 |
| hsa-miR-135b-5p | 0.15593011 | hsa-miR-135b-5p | 0.000053958 |
| hsa-miR-135a-5p | 0.129007803 | hsa-miR-135a-5p | 0.000040018 |
| hsa-let-7e-5p | 0.126783131 | hsa-let-7e-5p | 0.132722835 |
| hsa-let-7d-5p | 0.123724662 | hsa-let-7d-5p | 0.175904098 |
| hsa-miR-181b-5p | 0.108798551 | hsa-miR-181b-5p | 0.183381928 |
| hsa-let-7i-5p | 0.099645134 | hsa-let-7i-5p | 0.198676449 |
| hsa-miR-224-5p | 0.084296452 | hsa-miR-224-5p | 0.040178212 |
| hsa-miR-98-5p | 0.076260065 | hsa-miR-98-5p | 0.116515916 |
| hsa-miR-182-5p | 0.059845659 | hsa-miR-182-5p | 0.143892764 |
| hsa-miR-18b-5p | 0.045240105 | hsa-miR-18b-5p | 0.028237341 |
| hsa-miR-10a-5p | 0.042242151 | hsa-miR-10a-5p | 0.04290408 |
| hsa-miR-424-5p | 0.039192277 | hsa-miR-424-5p | 0.079408476 |
| hsa-miR-489-3p | 0.028225537 | hsa-miR-489-3p | 0.035895238 |
| hsa-miR-17-3p | 0.021616453 | hsa-miR-17-3p | 0.028388434 |
| hsa-miR-196b-5p | 0.018800731 | hsa-miR-196b-5p | 0.060667771 |
| hsa-let-7c-5p | 0.017275478 | hsa-let-7c-5p | 0.03305171 |
| hsa-miR-96-5p | 0.015125862 | hsa-miR-96-5p | 0.036364008 |
| hsa-miR-375 | 0.013888363 | hsa-miR-375 | 0.014864588 |
| hsa-miR-145-5p | 0.012514651 | hsa-miR-145-5p | 0.000996082 |
| hsa-miR-326 | 0.012146695 | hsa-miR-326 | 0.022353321 |
| hsa-miR-335-5p | 0.008966262 | hsa-miR-335-5p | 0.000040929 |
| hsa-miR-196a-5p | 0.007251265 | hsa-miR-196a-5p | 0.042092027 |
| hsa-miR-24-2-5p | 0.005609694 | hsa-miR-24-2-5p | 0.000948289 |
| hsa-miR-376a-3p | 0.003301945 | hsa-miR-376a-3p | 0.000040018 |
| hsa-miR-665 | 0.001043504 | hsa-miR-665 | 0.000741259 |
| hsa-miR-487b-3p | 0.00083316 | hsa-miR-487b-3p | 0.00025674 |
| hsa-miR-218-5p | 0.000675564 | hsa-miR-218-5p | 0.023301403 |
| hsa-miR-539-5p | 0.000653119 | hsa-miR-539-5p | 0.000069272 |
| hsa-miR-125b-5p | 0.000627544 | hsa-miR-125b-5p | 0.018692294 |
| hsa-miR-134-5p | 0.000501839 | hsa-miR-134-5p | 0.000558324 |
| hsa-miR-122-5p | 0.00047826 | hsa-miR-122-5p | 0.000040018 |
| hsa-miR-302a-3p | 0.000291654 | hsa-miR-302a-3p | 0.002410065 |
| hsa-miR-214-3p | 0.000214995 | hsa-miR-214-3p | 0.000544995 |
| hsa-miR-302b-5p | 0.00020397 | hsa-miR-302b-5p | 0.000124066 |
| hsa-miR-410-3p | 0.000189116 | hsa-miR-410-3p | 0.000134618 |
| hsa-miR-199a-3p | 0.000175368 | hsa-miR-199a-3p | 0.000373762 |
| hsa-miR-202-3p | 0.000140309 | hsa-miR-202-3p | 0.000472707 |
| hsa-miR-302d-3p | 0.00013828 | hsa-miR-302d-3p | 0.000131689 |
| hsa-miR-495-3p | 0.000133072 | hsa-miR-495-3p | 0.00023295 |
| hsa-miR-302c-3p | 0.000072690 | hsa-miR-302c-3p | 0.000266131 |
| hsa-miR-302a-5p | 0.000066140 | hsa-miR-302a-5p | 0.000112053 |
| hsa-miR-136-5p | 0.000048679 | hsa-miR-136-5p | 0.000040018 |
| hsa-miR-206 | 0.000048679 | hsa-miR-206 | 0.000040018 |
| hsa-miR-302c-5p | 0.000048679 | hsa-miR-302c-5p | 0.000070289 |
| hsa-miR-323a-3p | 0.000048679 | hsa-miR-323a-3p | 0.0000400183 |
| hsa-miR-367-3p | 0.00004.867 | hsa-miR-367-3p | 0.000040018 |
| hsa-miR-371a-5p | 0.000048679 | hsa-miR-371a-5p | 0.000040018 |
| hsa-miR-372-3p | 0.000048679 | hsa-miR-372-3p | 0.000072337 |
| hsa-miR-373-3p | 0.000048679 | hsa-miR-373-3p | 0.000040018 |
| hsa-miR-518b | 0.000048679 | hsa-miR-518b | 0.000141907 |
| hsa-miR-518c-3p | 0.000048679 | hsa-miR-518c-3p | 0.000040018 |
| hsa-miR-520a-3p | 0.000048679 | hsa-miR-520a-3p | 0.000183729 |
| hsa-miR-520b | 0.000048679 | hsa-miR-520b | 0.00014254 |
| hsa-miR-520e | 0.000048679 | hsa-miR-520e | 0.000040018 |
| hsa-miR-520g-3p | 0.000048679 | hsa-miR-520g-3p | 0.000130909 |
